# The anti-aflatoxigenic mechanism of cinnamaldehyde in *Aspergillus flavus*

**DOI:** 10.1038/s41598-019-47003-z

**Published:** 2019-07-19

**Authors:** Ping Wang, Longxue Ma, Jing Jin, Mumin Zheng, Lin Pan, Yueju Zhao, Xiulan Sun, Yang Liu, Fuguo Xing

**Affiliations:** 1 Institute of Food Science and Technology, Chinese Academy of Agricultural Sciences/Key Laboratory of Agro-products Quality and Safety Control in Storage and Transport Process, Ministry of Agriculture, Beijing, 100193 P.R. China; 2 State Key Laboratory of Food Science and Technology, School of Food Science, Synergetic Innovation Center of Food Safety and Nutrition, Jiangnan University, Wuxi, Jiangsu 214122 P.R. China

**Keywords:** Fungal genomics, Antifungal agents

## Abstract

Aflatoxin B_1_ (AFB_1_), the predominant and most carcinogenic naturally polyketide, is mainly produced by *Aspergillus flavus* and *Aspergillus parasiticus*. Cinnamaldehyde has been reported for inhibiting the growth and aflatoxin biosynthesis in *A. flavus*. But its molecular mechanism of action still remains largely ambiguous. Here, the anti-aflatoxigenic mechanism of cinnamaldehyde in *A. flavus* was investigated via a comparative transcriptomic analysis. The results indicated that twenty five of thirty genes in aflatoxin cluster showed down-regulation by cinnamaldehyde although the cluster regulators *aflR* and *aflS* were slightly up-regulated. This may be due to the up-regulation of the oxidative stress-related genes *srrA*, *msnA* and *atfB* being caused by the significant down-regulation of the diffusible factor *FluG*. Cinnamaldehyde also inhibited aflatoxin formation by perturbing GPCRs and oxylipins normal function, cell wall biosynthesis and redox equilibrium. In addition, accumulation of NADPH due to up-regulation of pentose phosphate pathway drove acetyl-CoA to lipids synthesis rather than polyketides. Both GO and KEGG analysis suggested that pyruvate and phenylalanine metabolism, post-transcriptional modification and key enzymes biosynthesis might be involved in the suppression of AFB_1_ production by cinnamaldehyde. This study served to decipher the anti-aflatoxigenic properties of cinnamaldehyde in *A. flavus* and provided powerful evidence for its use in practice.

## Introduction

*Aspergillus flavus*, as a widely distributed saprotrophic filamentous fungus especially in warmer and moister atmosphere, is the major safety problem in both agricultural and medical products^1^. It can produce an abundance of diverse secondary metabolites including aflatoxins, conidial pigments, cyclopiazonic acid, aflatrem and kojic acid^2,3^. Of them, aflatoxins are the predominant and most carcinogenic naturally occurring compounds which inevitably result in health complications, including hepatocellular carcinoma, acute intoxication, immune system disorder and growth retardation in children^4^. Therefore, aflatoxin remains a global threat to human and animal health, and is one of the key safety indicators of grain.

Many strategies have been used to reduce aflatoxin contamination. At present, chemical agents still are often used for controlling post-harvest aflatoxin contamination. However, these agents have many disadvantages such as toxicity, residues in food chain, and greater likelihood of resistance^5,6^. Therefore, facing with a huge burden and threat, people aroused the interest of discovering safe and efficient natural substances for preventing and controlling *A. flavus* growth and aflatoxin production. In previous studies, essential oils such as eugenol, carvacrol, citral and cinnamaldehyde, possessing potent anti-microbial, antioxidant, and other biological activities, were applied to food industry as food additive^7^. Cinnamaldehyde, a major component of Chinese cinnamon oil from *Cinnamomum spp*, is used as legally flavoring antimicrobial ingredient and referenced as “generally recognized as safe” for mankind and surroundings by the USFDA and FAO/WHO^8^. It has been widely used in food, booze to inhibit the growth of bacteria, yeast and filamentous fungi because of the wider spectrum antimicrobial activities since long time^9,10^. It was highly efficient for suppressing *Salmonella typhimurium* and *Staphylococcus aureus* in watermelon juice, and *Salmonella enterica* in apple juice^11^. Besides, growth of *Fusarium verticillioides*, *Aspergillus ochraceus*, *Penicillium expansum* and *A. flavus* has been remarkably inhibited by cinnamaldehyde^5,8,12,13^. In particular, it can depress the production of aflatoxin by *A. flavus*^1,8^. It also stimulates apoptosis and inhibits tumor growth^14^, and has been reported as an effective agent against several cancers effectively^15,16^.

Out of 10 essential oils previously studied by our group, cinnamaldehyde was the most effective against fungal growth and aflatoxin production by *A. flavus*^17^. Production of aflatoxin B_1_ (AFB_1_) was completely inhibited by cinnamaldehyde at lower concentration (0.4 mM) without influencing *A. flavus* growth, and at the concentration of 0.8 mM cinnamaldehyde showed complete inhibition of fungal growth and AFB_1_ production^1^. This was similar to the results reported by Sun^8^, which indicated that fungal growth and aflatoxin production were significantly inhibited by cinnamaldehyde in dose-dependent manner by modulating the oxidative stress in *A. flavus*. It takes inhibitory action against bacteria^11,18,19^, yeast and filamentous molds^20–22^ by depressing intracellular ATP^23^, cell wall biosynthesis^24^, and altering the membrane structure and integrity.

Using qPCR, Yin *et al*.^24^ found that cinnamaldehyde (0.005%) significantly inhibited AFB_1_ production in *A. flavus* and *A. parasiticus*. The expressions of the majority of aflatoxin gene cluster were down-regulated by more than 4-folds, especially *pksA* (*aflC*), *nor-1* (*aflD*), and *norA* (*aflE*). Our previous studies showed that AFB_1_ production was largely reduced in *A. flavus* treated with cinnamaldehyde at the low concentration in YES medium^1^. In the presence of cinnamaldehyde (0.4 mM), *aflM* was significantly down-regulated by more than 5963-fold, following by *aflP*, *aflR*, *aflD* and *aflT*. The decreased transcription levels of aflatoxin cluster genes subsequently resulted in the reduction of AFB_1_ production. Although many researchers develop desire at exploring the anti-aflatoxigenic mechanism of cinnamaldehyde, the detailed molecular mechanism behind in the inhibition of aflatoxin biosynthesis by cinnamaldehyde still remains largely ambiguous.

RNA-seq, a high-throughput sequencing technology used to sequence complementary DNA, has been applied to transcriptomic studies, including anti-fungi response mechanism to essential oils. Wang *et al*.^12^ found that cinnamaldehyde inhibited *P. expansum* by modulating the oxidative stress and down-regulating the ergosterol biosynthesis using transcriptional profiling analysis. In another report, the transcriptome profiling of *A. flavus* exposed to antioxidant gallic acid was used in exploring the response mechanism^25^. The gallic acid played a pivotal role in fungal development via over-expression of *brlA* while the velvet complex didn’t show a significant differential expression. In addition, other regulators were also involved in the inhibitory mechanism of gallic acid. In another transcriptional profiling analysis of *A. flavus* exposure to 5-azacytidine (5-AC), the up-regulation of *brlA* was also found^25^.

The main aim of this study was to investigate the role of cinnamaldehyde in the inhibition of fungal development and secondary metabolite biosynthesis of *A. flavus* via RNA-seq approach. The differentially expression genes between cinnamaldehyde treated and untreated *A. flavus* were obtained and further analyzed. Especially, the anti-aflatoxigenic mechanism of cinnamaldehyde was revealed. This work may also contribute to better understanding on the aflatoxin biosynthesis and regulation.

## Results

### Overall transcriptional response profile of *A. flavus* to natural cinnamaldehyde

To explore the latent detailed molecular mechanism response to natural cinnamaldehyde on *A. flavus*, a transcriptomes analysis was implemented to evaluate the response at mRNA level. Averagely, *A. flavus* YC-15 untreated and treated with cinnamaldehyde generated 10.63 million and 11.11 million raw reads, respectively. From these raw reads, 8.84 million and 9.26 million clean reads were obtained after purity filtering. And, 58.84% and 68.36% of total clean reads from control and treatment groups were mapped to the reference genome sequence while only 0.02% were aligned to rRNA genes. The mRNA data revealed that 1032 genes were significantly differentially transcribed between the *A. flavus* treated with cinnamaldehyde and the untreated sample. Among them, 427 genes’ transcripts showed up-regulation and 605 genes showed down-regulation in cinnamaldehyde-treated group compared with the untreated group.

### Functional classification and pathway analysis of differential expression genes (DEGs)

**Figure 1:**
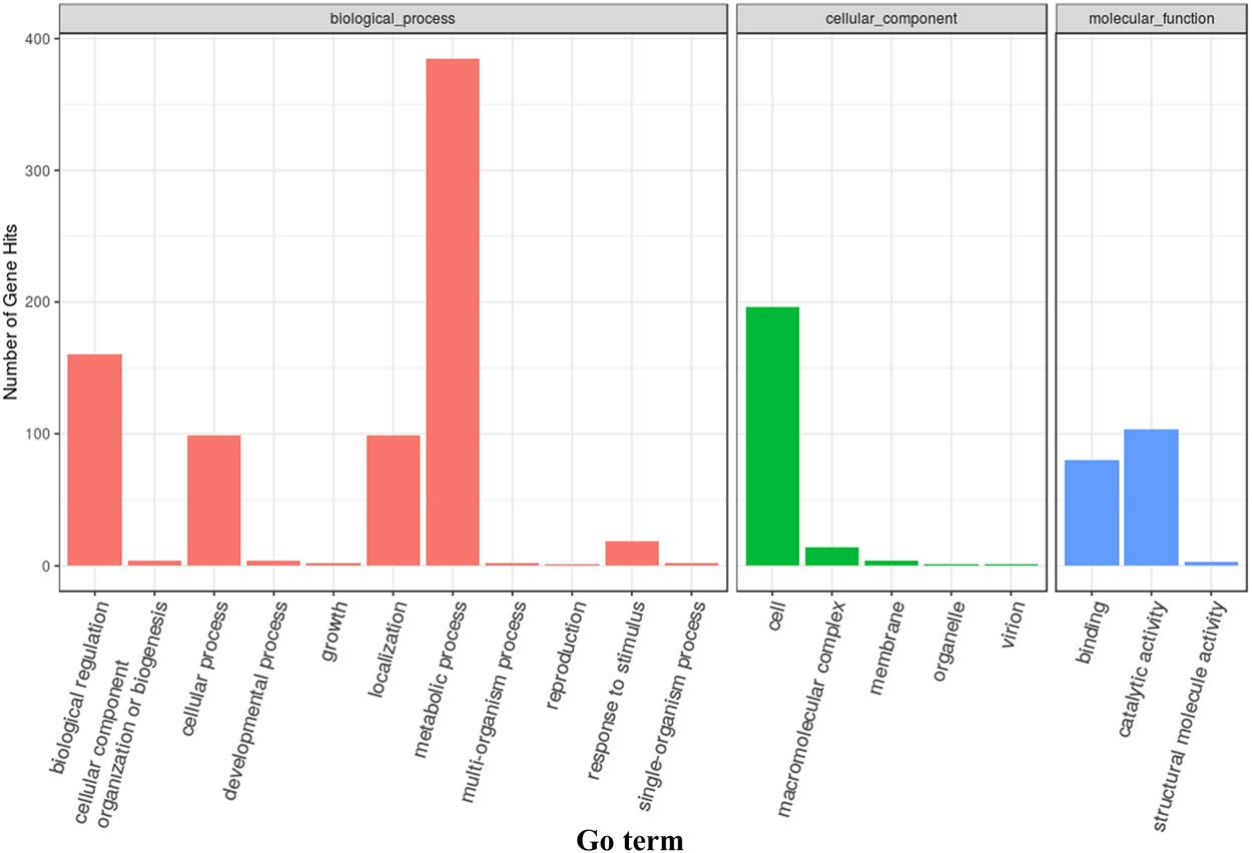
The gene ontology annotation of differential expression genes.

The DEGs between the *A. flavus* treated with cinnamaldehyde (R75) and control group (CK) provided a potential anti-aflatoxigenic mechanism of cinnamaldehyde related to *A. flavus*. These 1032 DEGs related to a large quantity of regulatory and metabolic process were identified (with FDR ≤ 0.05, log_2_Ratio ≥1 or ≤1) between R75 and CK according to the FPKM values. In order to analyze the functions of 1032 DEGs, GO functional and KEGG metabolic pathways enrichment analyses were performed. GO analysis revealed that these significantly DEGs were mainly involved in oxidoreductase activity, RNA binding, Nuclease activity and translation ignition factor activity (Table 1, Fig. 1). KEGG analysis revealed that these significantly DEGs were mainly involved in RNA transport, ribosome biogenesis, pyruvate metabolism, phenylalanine metabolism, sulfur relay system and sulfur metabolism (Table 2).

**Table 1:** GO functional enrichment analysis of DEGs when *A. flavus* was treated with cinnamaldehyde.

GO ID	Description	*p*-value	*q*-value	List hits
GO:0003723	RNA binding	8.31E-03	2.07E-01	14/149
GO:0016614	oxidoreductase activity, acting on CH-OH group of donors	2.47E-02	4.32E-01	9/149
GO:0004518	nuclease activity	1.84E-02	3.56E-01	6/149
GO:0003743	translation initiation factor activity	1.43E-03	2.07E-01	5/149

**Table 2:** KEGG metabolic pathway enrichment analysis of DEGs when *A. flavus* was treated with cinnamaldehyde.

ID	TERM (molecular functions)	*p*-value	*q*-value	List hits
afv00620	Pyruvate metabolism	0.0174	0.286941	8/58
afv00360	Phenylalanine metabolism	0.0794	0.785848	7/58
afv04122	Sulfur relay system	0.0302	0.373098	4/58
afv03013	RNA transport	0.0016	0.040238	3/58
afv03008	Ribosome biogenesis in eukaryotes	0.0010	0.040238	2/58
afv00920	Sulfur metabolism	0.0980	0.808409	2/58

### Genes involved in biosynthesis of conidial pigment, aflatrem, aflatoxin and cyclopiazonic acid

**Figure 2:**
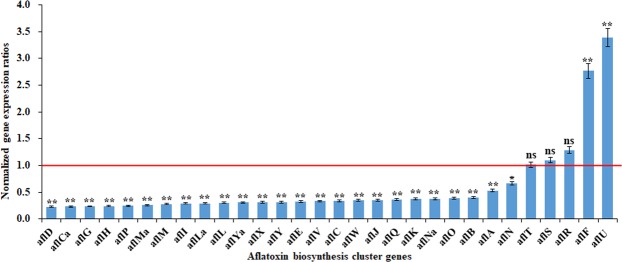
Expression ratios of genes in the aflatoxin biosynthesis cluster in response to 0.60 mM of cinnamaldehyde. Red line represents control expression level. ns = not significant; *p < 0.05; **p < 0.01.

The expression profile referred to the biosynthesis of conidial pigment (#10), aflatrem (#15), aflatoxin (#54), and cyclopiazonic acid (#55) were evaluated and shown in Table 3. In pathway #10, O-methyltransferase family protein (AFLA_016120) and a hypothetical protein (AFLA_016130) were down-regulated, but *arp1* gene was up-regulated. In pathway #15, the majority of cluster genes showed low-level expression. In pathway #55, MFS multidrug transporter (AFLA_139460) was slightly down-regulated, nevertheless the genes encoding a hybrid PKS/NRPS enzyme (AFLA_139490), FAD dependent oxidoreductase (AFLA_139470), and tryptophan dimethylallyltransferase (AFLA_139480) were up-regulated. Our previous studies confirmed that cinnamaldehyde can repress the aflatoxin production and development in dose-dependent manner^1^. Based on the transcriptome data, 25 of 34 genes in aflatoxin biosynthetic gene cluster were down-regulated to varying degrees including the key structural genes *aflC*, *aflD*, *aflE*, *aflG*, *aflH*, *aflI*, *aflL*, *aflM*, *aflO*, *aflP* and *aflQ* in *A. flavus* treated with 0.60 mmol/L of cinnamaldehyde. Surprisingly, both transcription regulator genes *aflR* and *aflS* in aflatoxin cluster showed a slight up-regulation. However, the lower level of *aflS/aflR* ratio was observed compared with the untreated group, subsequently resulting in the down regulation of most structural genes. The *aflU* gene, encoding a P450 monooxygenase and probably involving in the biosynthesis of AFG_1_ and AFG_2_ rather than AFB_1_, was up-regulated after treatment of cinnamaldehyde. The *aflT* gene, encoding a membrane-bound protein presumed to be involved in aflatoxin secretion, was not affected by cinnamaldehyde. Furthermore, genes involved in aflatoxin biosynthesis pathway were analyzed through qPCR and the results were shown in Fig. 2. Meantime, the sugar cluster genes *sugR*, *orf*, *glcA* and *hxtA* were significantly up-regulated by cinnamaldehyde.

**Table 3:** The expression levels of genes in the biosynthesis of conidial pigment (#10), aflatrem (#15), aflatoxin (#54) and cyclopiazonic acid (#55).

Cluster ID	Gene ID (AFLA_x)	Untreated (FPKM)	R75 (FPKM)	LOG	Annotated gene function
#10	016120	10.58	5.31	−0.99	O-methyltransferase family protein
#10	016130	13.25	6.91	−0.94	hypothetical protein
#10	016140	14.10	20.44	0.54	conidial pigment biosynthesis scytalone dehydratase Arp1
#15	045450	37.27	47.33	0.34	ankyrin repeat-containing protein, putative
#15	045460	1.16	2.50	1.11	hypothetical protein
#15	045470	0.10	0.07	−0.43	nonsense-mediated mRNA decay protein, putative
#15	045480	0.32	1.35	2.08	conserved hypothetical protein
#15	045490	0.03	0.16	2.26	dimethylallyl tryptophan synthase, putative
#15	045500	0.55	0.26	−1.10	cytochrome P450, putative
#15	045510	0.12	0.07	−0.89	integral membrane protein
#15	045520	0.00	0.00	/	integral membrane protein
#15	045530	0.23	0.00	down	conserved hypothetical protein
#15	045540	0.00	0.00	/	cytochrome P450, putative
#15	045550	1.24	2.57	1.05	hypothetical protein
#15	045560	1.94	3.00	0.63	carboxylic acid transport protein
#15	045570	1.55	0.07	−4.42	acetyl xylan esterase, putative
#54	139390	231.56	105.85	−1.13	*aflD*/*nor-1*/reductase
#54	139400	84.14	39.09	−1.11	*aflCa*/ *hypC* /hypothetical protein
#54	139260	48.69	23.00	−1.08	*aflG*/ *avnA*/ *ord-1*/cytochrome P450 monooxygenase
#54	139330	192.79	92.60	−1.06	*aflH*/ *adhA*/short chain alcohol dehydrogenase
#54	139210	92.70	45.79	−1.02	*aflP*/*omtA*/*omt-1*/O-methyltransferase A
#54	139290	136.29	69.97	−0.96	*aflMa*/*hypE*/hypothetical protein
#54	139300	496.53	274.75	−0.85	*aflM*/*ver-1*/dehydrogenase/ketoreductase
#54	139230	15.55	9.00	−0.79	*aflI*/*avfA*/cytochrome P450 monooxygenase
#54	139240	108.16	63.18	−0.78	*aflLa*/*hypB*/hypothetical protein
#54	139250	92.87	56.46	−0.72	*aflL*/*verB*/desaturase/P450 monooxygenase
#54	139140	5.53	3.38	−0.71	*aflYa*/*nadA*/NADH oxidase
#54	139160	117.97	73.17	−0.69	*aflX*/*ordB*/monooxygenase/oxidase
#54	139150	101.03	63.54	−0.67	*aflY*/*hypA*/*hypP*/hypothetical protein
#54	139310	180.51	116.23	−0.64	*aflE*/*norA*/*aad*/*adh-2*/NOR reductase/dehydrogenase
#54	139180	48.91	32.57	−0.59	*aflV*/*cypX*/cytochrome P450 monooxygenase
#54	139410	37.55	25.15	−0.58	*aflC* /*pksA*/*pksL1*/polyketide synthase
#54	139170	49.07	34.09	−0.53	*aflW*/*moxY*/monooxygenase
#54	139320	132.91	94.02	−0.50	*aflJ*/*estA*/esterase
#54	139200	12.91	9.38	−0.46	*aflQ*/*ordA*/*ord-1*/oxidoreductase/cytochrome P450 monooxigenase
#54	139190	112.43	83.35	−0.43	*aflK*/*vbs*/VERB synthase
#54	139270	572.31	434.92	−0.40	*aflNa*/*hypD*/hypothetical protein
#54	139220	187.22	143.20	−0.39	*aflO*/*omtB*/*dmtA*/O-methyltransferase B
#54	139370	35.31	28.44	−0.31	*aflB* /*fas-1*/fatty acid synthase beta subunit
#54	139380	19.45	15.90	−0.29	*aflA*/*fas-2*/*hexA*/fatty acid synthase alpha subunit
#54	139280	34.04	31.21	−0.13	*aflN/verA*/monooxygenase
#54	139420	100.86	102.36	0.02	*aflT/aflT*/transmembrane protein
#54	139340	177.63	195.04	0.13	*aflS*/ pathway regulator
#54	139360	64.90	83.53	0.36	*aflR* / *apa-2/afl-2* / transcription activator
#54	139440	14.48	20.02	0.47	*aflF / norB*/dehydrogenase
#54	139110	2.38	3.49	0.55	*aflYd*/ sugR/sugar regulator
#54	139100	2.96	4.78	0.69	*aflYe/orf*/Ser-Thr protein phosphatase family protein
#54	139430	20.76	35.15	0.76	*aflU/cypA*/P450 monooxygenase
#54	139120	1.85	3.52	0.93	*aflYc/glcA*/glucosidase
#54	139130	1.78	3.93	1.15	*aflYb/hxtA*/putative hexose transporter
#55	139460	1293.63	1202.44	−0.11	MFS multidrug transporter, putative
#55	139470	215.54	687.52	1.67	FAD dependent oxidoreductase, putative
#55	139480	243.62	522.50	1.10	tryptophan dimethylallyltransferase
#55	139490	9.14	32.13	1.81	hybrid PKS/NRPS enzyme

### Genes involved in fatty acids *β*-oxidation and pentose phosphate pathway

Fatty acids *β*-oxidation in peroxisome and mitochondria promoted aflatoxin formation^26^. And there is a competition in acetyl-CoA between lipid synthesis and polyketides formation^27^. The transcriptional levels related fatty acids *β*-oxidation and pentose phosphate pathway were shown in Table S1. A large number of fatty acids *β*-oxidation-related genes were significantly down-regulated. The most strongly down-regulated gene in peroxisome was AFLA_009410, followed by AFLA_135240, AFLA_091060, and AFLA_090720. However, most of the genes in pentose phosphate pathway were up-regulated in *A. flavus* exposure to cinnamaldehyde, for example, *Zwf1* (AFLA_086620), *Sol* (3AFLA_080390), and *Gnd1* (AFLA_036840).

### Genes involved in ergosterol biosynthesis

The plasma membrane plays a crucial role in maintaining homeostasis, exchanging materials, and transduction of information. And ergosterol is one key constituent of fungal membrane^19,28^. The transcriptional levels related ergosterol pathway was shown in Table S2. Transcriptional levels of several genes were down-regulated after cinnamaldehyde treatment, for example, sterol delta 5,6-desaturase Erg3 (AFLA_018090), squalene monooxygenase Erg1 (AFLA_061500), and C-14 sterol reductase (AFLA_051080, AFLA_111350).

### Genes involved in fungal development

The regulation of secondary metabolism is associated with fungal growth and development. From the expression profile data, we found that the expression patterns of some gene referred to conidiophores development were down-regulated when *A. flavus* was treated with cinnamaldehyde (Table S3). For the velvet complex, *veA* did not show a significant differential expression while *velB*, *leaA*, and *vosA*, were slightly up-regulated exposure to cinnamaldehyde. *fluG* (AFLA_039530), encoding a protein comprising an N-terminal amidohydrolase domain and a C-terminal glutamine synthetase domain^29^, was down-regulated. And *esdC*, an early sexual development gene, was mildly down-regulated. Nevertheless, development regulator *FlbA* was up-regulated. *BrlA* mediating conidiophores, and *AbaA* controlling phialide differentiation were also up-regulated. In addition, *RodA* and *RodB*, conidial hydrophobic genes, both showed strong up-regulation.

### Genes involved in oxidative stress

In *A. flavus*, transcriptional factors AtfA, AtfB, AP-1, and MsnA are related to oxidative stress and aflatoxin biosynthesis. And oxylipin synthesis mediates oxidative processes and aflatoxin formation. The expression levels concerning oxidative stress related genes are shown in Table S4. Among the 47 relevant genes, *ap-1*, *atfB* and *msnA* were all up-regulated. The cellular receptors *gprC*, *gprF*, *gprK*, *gprM*, *gprR*, *gprP*, *gprS*, the oxylipins *ppoA*, *ppoB* and *ppoC*, the MAP kinase genes *mkk2*, *fus3*, *pbs2*, *mpkA*, *sakA*, *bck1*, *ste11*, *sskB* and *ste7*, and catalase gene *cat1*, *catA*, and superoxide dismutase gene *sod1*, *mnsod* were all up-regulated to varying degrees. *AfPXG*, encoding calcium binding protein caleosin, and GPCRs (*gprA*, *gprB*, *gprD*, *gprG*, *gprH*) were all down-regulated.

## Discussion

Cinnamaldehyde is gradually regarded as safer food additive in food processing and manufacturing comparing to chemical fungicides. The inhibitory effects and mechanism of cinnamaldehyde on fungal growth and mycotoxin have been reported by many researchers^1,8^. In our previous study, 0.4 mM cinnamaldehyde inhibited AFB_1_ production with the rate of 68.9%, and 0.8 mM cinnamaldehyde could completely suppress *A. flavus* growth^1^. In this study, the mechanism of *A. flavus* growth and aflatoxin formation dysfunction exposure to cinnamaldehyde was investigated by RNA-seq analysis. Moreover, the anti-fungal and anti-aflatoxigenic properties of cinnamaldehyde were discussed and conclusions were drawn based on the results of the previous studies and this study.

Aflatoxin synthesis is supported by the action of enzymatic cascade and involves 21 steps^30^. In *A. flavus*, this process is managed by a gene cluster in which *aflR* and *aflS* serve as regulators^31,32^. In our RNA-seq data, 25 genes of the aflatoxin biosynthesis cluster were down-regulated after treatment with cinnamaldehyde although *aflR* and *aflS* were up-regulated. With the exception of an up-regulated result for *aflF*, all the structural genes in the cluster were down-regulated. However, none of these was completely suppressed. The most strongly down-regulation gene was *aflD*, followed by the key structural genes *aflG*, *aflH*, *aflP*, *aflM*, *aflI*, *aflL* and *aflE*. The expression levels of all genes in the cluster were confirmed by q-PCR (Fig. 2). The *aflF*, encoding a dehydrogenase, is involved in the conversion of NOR to AVN^33^. The expression level of *aflF* was up-regulated, but its two homology protein genes *aflD* and *aflE* both were down-regulated. The gene *aflT*, encoding a membrane-bound protein presumed to be involved in aflatoxin secretion, was not modulated after treatment with cinnamaldehyde. And similar results were also reported in *A. flavus* treated with piperine^34^ and eugenol^34,35^. These findings indicated that cinnamaldehyde suppressed aflatoxin biosynthesis by down-regulating the transcript levels of most structural genes.

An astonishing result is that the transcriptional factor *aflR* and cofactor *aflS* showed a mild up-regulation in *A. flavus* treated with cinnamaldehyde. In our previous study^35^, the expression level of *aflR* and *aflS* showed slight up-regulation in *A. flavus* treated with eugenol although aflatoxin production was significantly inhibited by eugenol. Similarly, 5-Azacytidine (5-AC) suppressed fungal development and aflatoxin synthesis while *aflR* and *aflS* were mildly up-regulated^36^. The results are also much like with the findings reported by Zhao and his colleagues^25^. They found that *aflR* and *aflS* were up-regulated slightly while structure gene showed down-regulation exposure to an antioxidant gallic acid which could inhibit *A. flavus* development and aflatoxin production. The similar result that the aflatoxin cluster regulators *aflR* and *aflS* were up-regulated slightly although most structural genes were down-regulated in *A. flavus* treated with different anti-aflatoxigenic natural compounds, suggesting the stable expression of *aflR* and *aflS*.

The velvet complex was critical for conidiation and aflatoxin formation in *A. flavus*^37,38^. In the deletion mutant of *veA*, the expression of key aflatoxin genes including *aflR*, *aflD*, *aflM* and *aflP* was completely suppressed. Consequently, aflatoxin was halted^39^. However, *veA* did not show significant differential expression although almost all structural genes were down-regulated. The oxidative stress-related genes such as *msnA*, *srrA*, *atfB* and *pacC*, which were positively regulated by *veA*, were up-regulated after cinnamaldehyde treatment. The similar result was obtained in *A. flavus* treated with eugenol^35^. The *LaeA* and *velB* genes, encoding the other two proteins of velvet complex, were slightly up-regulated. Interestingly, a velvet-related gene *FluG* were significantly down-regulated in *A. flavus* treated with cinnamaldehyde. FluG, composed of an N-terminal amidohydrolase domain and C-terminal glutamine synthetase domain, was assumed for synthesizing a diffusible factor^40^. Chang *et al*. (2013) reported that *VeA*, *VelB*, and *LaeA*, combined with *FluG*, were indispensable to maintaining conidiation program, sclerotial formation, and aflatoxin biosynthesis in *A. flavus*^41^. These results suggested that *FluG* may play an important role in the anti-aflatoxigenic mechanism by cinnamaldehyde.

Acetyl-CoA, the fundamental structure element of all known fungal polyketides, is mainly produced from fatty acids *β*-oxidation and glycolysis of sugars. For aflatoxin biosynthesis, fatty acids β-oxidation is a major contributor to acetyl-CoA^26^. It was reported that pentose phosphate pathway activity was associated with lower content of aflatoxin. Zhao *et al*.^25^ found that gallic acid inhibited the aflatoxin formation via up-regulation of pentose phosphate pathway. Incubated in aflatoxin inhibitory medium, *A. flavus* pentose phosphate pathway was accelerated leading to NADPH accumulation^42^. Ultimately, acetyl-CoA was converted into lipid biosynthesis rather than polyketide formation^27^. In our data, there were large number of fatty acids β-oxidation-related genes showing significant down-regulation after cinnamaldehyde treatment, such as AFLA_019280 (peroxiredoxin), AFLA_052400 (isocitrate lyase) and AFLA_009410 (delta (3,5)-delta (2,4)-dienoyl-CoA isomerase) (Table S3). Meantime, most of the genes involved in pentose phosphate pathway were up-regulated exposure to cinnamaldehyde including AFLA_041580 (estradiol 17 beta-dehydrogenase), AFLA_115890 (acyl-CoA oxidase) and AFLA_080390 (6-phosphogluconolactonase Sol). These results suggested that the down-regulation of fatty acids *β*-oxidation and the up-regulation of pentose phosphate pathway were also associated with the anti-aflatoxigenic mechanism of cinnamaldehyde.

Cinnamaldehyde was considered to make its antifungal effects on perturbing cell wall biosynthesis, ergosterol biosynthesis and ATPase^43^. The 4 genes associated with cell wall, AFLA_098380, AFLA_083360, AFLA_014260 and AFLA_100100, were down-regulated. The similar phenomenon had been reported that cinnamaldehyde caused several genes involved in cell wall biosynthesis dysfunction^12,24^. Ergosterol is one of the principal sterol ingredients in the fungal membrane and is crucial for survival due to the ability in maintaining cell membrane fluidity, permeability, and pheromone signaling^44–46^. In the present work, the transcriptional level of several genes related ergosterol was down-regulated, for example squalene monooxygenase *Erg1* (AFLA_061500), C-14 sterol reductase (AFLA_051080 and AFLA_111350). The *Erg1* gene of *S. cerevisiae* encodes squalene epoxidase, a key enzyme in the ergosterol pathway. Disruption of the gene resulted in a lethal phenotype when cells grew under aerobic conditions, even in the presence of ergosterol^47^. C-14 sterol reductase (AFLA_051080 and AFLA_111350) were all down-regulated. Double deletion of *Erg25* genes was lethal in *A. fumigatus*^48^. Cinnamaldehyde weakened ergosterol biosynthesis which resulted in the disruption of the intracellular ATP, and some essential irons equilibrium^43^. In *E. coli* and *Listeria monocytogenes*, cinnamaldehyde inhibited the membrane-bound ATPase activity^49,50^. In the present study, some genes related to mitochondrial ATPase activity were repressed, for example, mitochondrial F1F0 ATP synthase subunit (AFLA_129660, AFLA_032070 and AFLA_043330).

RNA-binding was found to be the most dysregulated function after cinnamaldehyde treatment using GO enrichment analysis. Our previous study found the similar results in *A. flavu*s treated with eugenol^35^. Therefore, similar with eugenol, the post-transcriptional regulation may play an important role in the anti-aflatoxigenic mechanism of cinnamaldehyde. KEGG metabolic pathway analysis showed that pyruvate metabolism and phenylalanine metabolism were the main dysregulated metabolic pathway after cinnamaldehyde treatment. Pyruvate locates intersection of intermediary metabolism, which refers to multiple metabolic processes covering gluconeogenesis, lipogenesis and energy production^51^. As a metabolic switch, the pyruvate dehydrogenase complex (PDH) was considerable for carbon metabolism because of turning pyruvate into acetyl-coA^52^. Acetyl-CoA and malonyl-CoA are precursor substances in aflatoxin formation^53^. Besides, PDH was crucial for morphology and pathogenicity in different fungal species^54,55^. Amino acid metabolism plays an important role in aflatoxin biosynthesis. It was reported that phenylalanine metabolism was dysregulated in *A. flavus* treated with 2-phenylethanol^56^. In addition, phenylalanine was lightly incorporated into aflatoxin in *A. flavus*^57^. These results suggested that pyruvate metabolism and phenylalanine metabolism dysfunction might result in the reduction of aflatoxin biosynthesis.

Different stress can perturb cellular redox equilibrium, resulting in enhancive reactive oxygen species (ROS) levels named oxidative stress^58^. Excessive accumulation of ROS can jeopardize DNA, proteins and lipids, leading to cellular dysfunction^59^. Several researchers have thought that oxidative stress is a pre-condition for aflatoxin biosynthesis in *A. flavus* and *A. parasticus*^60,61^. The hypothesis is associated with the tentative that aflatoxin biosynthesis protects the fungus against oxidative stress. Reverberi *et al*.^59^ introduced a *P33* gene into *A. flavus* resulting in enhanced ROS accompanying aflatoxin accumulation. On the contrary, antioxidants such as gallic acid and ethylene reduced the oxidative stress in *A. flavus* leading to the decrease of aflatoxin content^25^. GPCRs and oxylipins are tied in oxidative process. The expression levels in regard to oxidative-related genes were shown in Table S3. After cinnamaldehyde exposure, 7 GPCRs and 2 oxylipins genes showed significant differential expression. In this study, we found that *gprC*, *gprF*, *gprK, gprM* and *grpS* were significantly up-regulated with AFB_1_ inhibition in *A. flavus* treated with cinnamaldehyde. Similar results were obtained in *A. flavus* treated with eugenol in our previous study^35^. The genes, *grpC*, *gprF*, *gprK*, *gprM* and *gprS* were also up-regulated after eugenol treatment. Caceres *et al*.^34^ also reported that over-expressed *gprK* accompanied with lower content of AFB_1_. Oxylipins pathway includes four genes, *ppoA*, *ppoB*, *ppoC*, and *afPXG* in *A. flavu*s^62^. Affeldt *et al*.^63^ reported that high content of oxylipins was associated with lower levels of aflatoxins. Simultaneous silencing via RNAi of *ppoA*, *ppoB* and *ppoC* and *AfPXG* resulted in an increase of aflatoxin biosynthesis^62^. Caceres *et al*.^34^ also found that over expression of *ppoB* and *ppoA* was correlated with AFB_1_ inhibition by piperine. In present study, the expression levels of *ppoA*, *ppoB* and *ppoC* were all up-regulated, suggesting the decreased oxylipins genes expression was associated with AFB_1_ inhibition by cinnamaldehyde. All these results suggest that the up-regulation of GPCRs and oxylipins genes was involved in AFB_1_ inhibition by cinnamaldehyde.

In *A. flavus* and *A. parasiticus*, there were several *bZIP* transcription factors referring to aflatoxin biosynthesis and oxidative stress response. Among these, SrrA, AtfB, AP-1, and MsnA were characterized as co-regulators^60,64–67^. In this study, we found that genes belonging to bZIP-type family were involved in the anti-aflatoxigenic mechanism of cinnamaldehyde. SrrA, an orthologue of *S. cerevisiae* Skn7 and *Saccharomyces pombe* Prr1, controlled key functions in response to osmotic and oxidative stress and was considered as a regulator in aflatoxin biosynthesis^64^. AP-1, a highly conserved protein in mammalian, yeast and fungi^60,68,69^. AP-1 may play crucial roles in sensing ROS because of high cysteine content in N- and C-terminal^70^. Over-expression of *napA*, an ortholog of *AP-1*, resulted in secondary metabolite inhibition in *A. nidulans* which implied napA was a negative regulators in secondary metabolite synthesis^71^. In *A. parasiticus*, the *ApyapA* disruption resulted in more aflatoxin production^60,65^. In this study, the *ap-1* showed up-regulation accompanying with aflatoxin inhibition in *A. flavus* cinnamaldehyde exposure. Similar results were also obtained by Caceres *et al*.^34^. They found that the *AP-1* was up-regulated with aflatoxin inhibition in *A. flavus* after piperine treatment. *AtfA* mediates several processes in vegetative hyphae, contributes to stress tolerance and changes secondary metabolism in *A. nidulans*^72^, *A. oryzae*^73^, and *A. fimugatus*^74^. *AtfB*, an orthologue of *AtfA*, is an important regulator referring to aflatoxin production and oxidative stress via binding to CER sites of aflatoxin biosynthesis genes promoter^64,67^. This CRE binding site was found in 7 genes promoter regions^34^. In the present study, *AtfA* did not show significant differential expression while *AtfB* was up-regulated by cinnamaldehyde. Caceres *et al*.^34^ also found *AtfB* was up-regulated with decreased production of aflatoxin after piperine treatment. *MsnA* has an important effect on fungal growth, aflatoxin and kojic acid formation, and oxidative stress^64^. In *A. flavus* and *A. parasiticus*, *MsnA* disruption resulted in aflatoxin and ROS accumulation^75^. In our previous study, we also found that transcript factor MsnA played a negative role in aflatoxin biosynthesis^35^. Similar result was obtained in *A. flavus* treated with cinnamaldehyde. Taken together, *srrA*, *atfB*, *ap-1*, and *msnA* were all up-regulated after cinnamaldehyde exposure. These results implied that *bZIP* transcription factors SrrA, AtfB, AP-1, and MsnA up-regulation played a direct negative role in aflatoxin formation after cinnamaldehyde treatment.

Antioxidant enzymes SOD and CAT which were regulated by the bZIP transcription factors make crucial effect on defense against ROS^12^. Many publications have reported that some inhibitors could suppress aflatoxin formation via positive regulating the antioxidant enzymes activities. However, different aflatoxin inhibitors act on different type of antioxidant enzymes. For example, piperine and β-glucans from *Lentinula edodes* led to lower AFB_1_ production with higher CAT activity^34^. Oppositely, eugenol and ascorbic acid sharply depressed the AFB_1_ biosynthesis accompanying with high SOD activity^34^. In addition, gallic acid may equilibrium ROS by activating the glutathione- and thioredoxin-dependent antioxidant system instead of changing CAT and SOD activities^25^. In this study, we found that antioxidant enzymes catalase gene (*cat*, *cat1*, and *catA*), and superoxide dismutase gene (*sod1*, and *mnSOD*) were all up-regulated in *A. flavus* treated with cinnamaldehyde. However, Sun *et al*.^8^ reported that exposure to cinnamaldehyde only resulted in higher SOD activity using the hydroxylamine analysis. The different results may imply that (1) reveals a dose effect; (2) exists a post-translational modification of CAT. These results made it clear that cinnamaldehyde enhanced CAT and SOD activities as part of its anti-aflatoxigenic mechanism.

**Figure 3:**
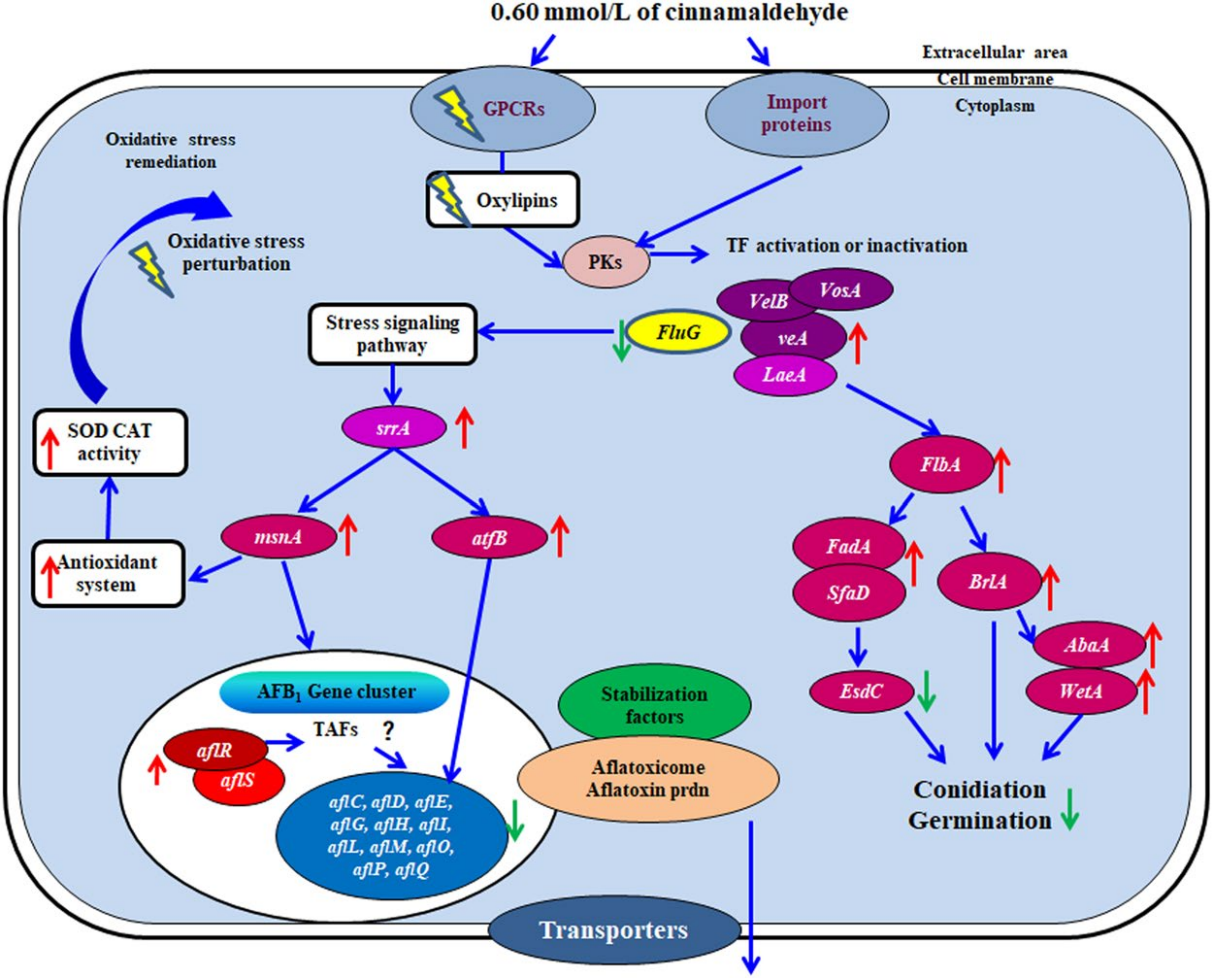
Hypothetical mechanism of action of cinnamaldehyde. Up- or down-regulation of gene on cinnamaldehyde exposure is represented using red and green arrow. PKs, protein kinase; TF, transcription factor.

Figure 3 shows the hypothetical gene modulation mode of action on aflatoxin formation and fungal growth in *A. flavus* treated with cinnamaldehyde at transcription levels. The signal transduction disorder happens when cinnamaldehyde regulates the expression of GPCRs and oxylipins genes. Velvet complex together with FluG modulates conidiation, sclerotial production, and aflatoxin biosynthesis. However, the differential expression of *LaeA*, *veA*, and *VelB* was not significant. The down-regulation of *FluG* may trigger the expression of stress response transcriptional factor gene *srrA*, which results in up-regulation of bZIP transcriptional factor *ap-1*, zinc finger transcriptional factor *msnA*, and CREB/ATF family member *atfB*. Ultimately, the redox system is perturbed and then antioxidant enzymes are activated. In addition, AP-1, MsnA, AtfB, as negative regulatory factors, modulate aflatoxin biosynthesis gene cluster. For conidia development, early asexual development factor FlbA is modulated by velvet complex and FluG. Up-regulation of *FlbA* activates *FadA* and *SfaD* which play a negative role in the expression of *esdC*. Besides, *FlbA* causes the up-regulation of *BrlA* which triggers over-expression of *AbaA* and *wetA*. Taken together, down-regulation of *esdC* and over-expression of *BrlA*, *AbaA*, and *wetA* facilitate asexual development.

**Figure 4:**
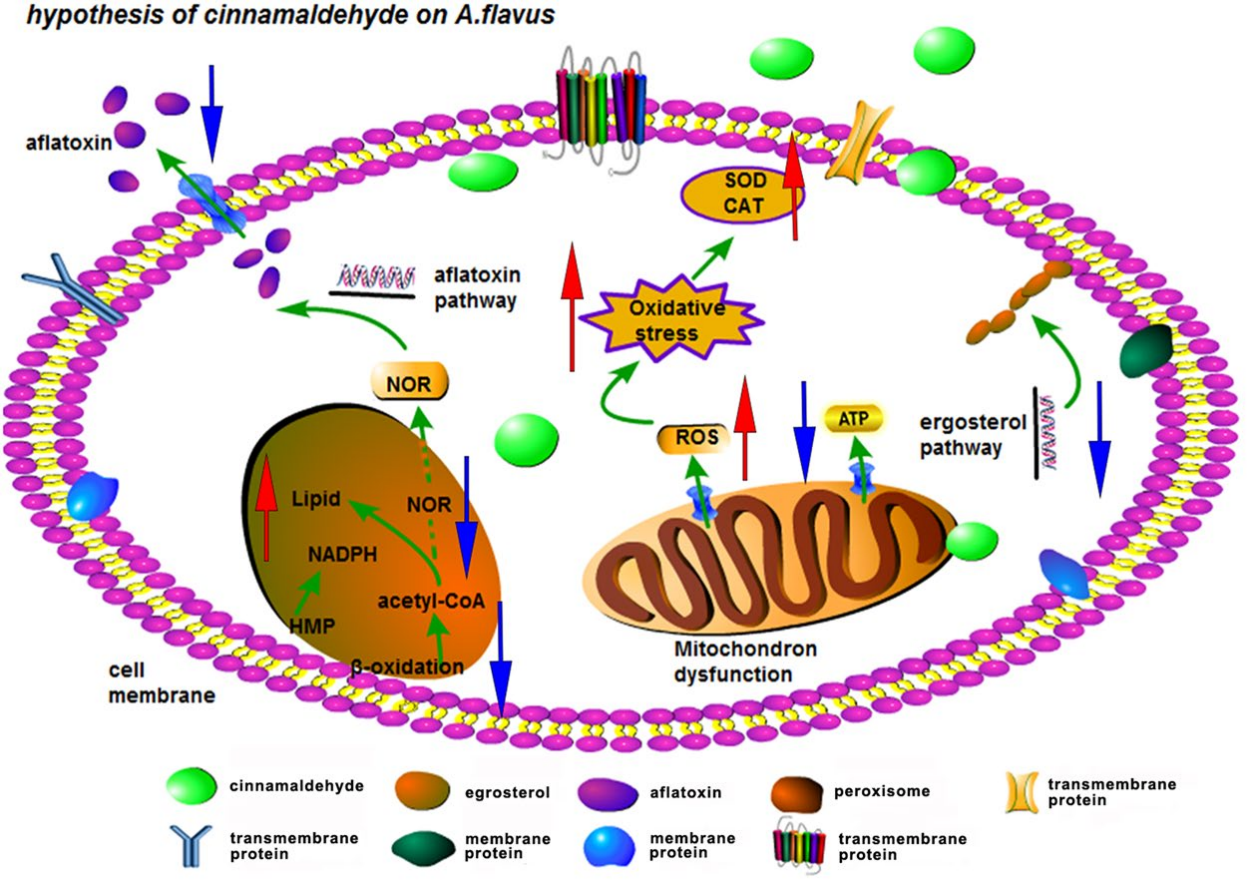
An elementary diagram elucidating the antifungal effect of cinnamaldehyde on *A.flavus* YC-15.

To sum up in Figs 3 and 4, cinnamaldehyde inhibits the aflatoxin biosynthesis and fungal growth of *A. flavus* via (1) reducing the fatty acid oxidation level by modulating several oxidation-related genes which leads to marked reduction of aflatoxin precursor acetyl-CoA; (2) increasing the NADPH accumulation by HMP which competes with aflatoxin biosynthesis; (3) weakening ergosterol synthesis which does damage to cell membrane integrity accompanied with altering the intracellular ATP and some indispensable iron equilibrium; (4) disturbing the redox system and then activating antioxidant enzymes which are deemed as key elements for regulating aflatoxin-related genes. These results uncovered in this study play a critical role in understanding the anti-aflatoxigenic mechanism of cinnamaldehyde in *A. flavus* and may accelerate its use in practice. Moreover, these results should assist further studies on the mechanism of action of inhibitor against fungal growth and mycotoxin production.

## Conclusion

The results of this study put forward a mechanism to explain the transcription regulation concerning the inhibitory effect of cinnamaldehyde on aflatoxin biosynthesis via RNA-seq. On basis of early studies, we draw a conclusion that (1) the decline in aflatoxin biosynthesis is on account of the down-expression of most of structural genes of aflatoxin cluster after treatment with cinnamaldehyde; (2) accumulation NADPH drives acetyl-CoA to lipid synthesis rather than polyketide formation; (3) the down-expression of diffusible factor *FluG* working with the velvet complex and the concomitant up-regulation of the oxidative stress-related genes *srrA*, *msnA*, and *atfB*; (4) dysfunction of GPCRs and oxylipins genes; (5) post-transcriptional modification and key enzymes biosynthesis may be involved in the suppression of AFB_1_ formation by cinnamaldehyde.

## Materials and Methods

### Natural compound, strain, and growth conditions

Natural cinnamaldehyde (99%) was purchased from Jiangxi Xue Song Natural Medicinal Oil Co., Ltd. (Ji’an City, Jiangxi, China). The strain *A. flavus* YC-15^35^ was inoculated in PDA medium (200 g boiled potato, 20 g dextrose, 20 g agar, 1 L) in the dark. The conidia from a PDA culture grown for 7 d at 28 °Cwere washed with 0.01% Tween-20 solution, counted and added into YES liquid medium (20 g yeast extract, 150 g sucrose, 0.5 g MgSO_4_·7H_2_O, 1 L) at a final concentration of 10^6^ conidia/mL. The cinnamaldehyde was added into the YES cultures at a final concentration of 0.60 mM. As the control group, cinnamaldehyde was absent. All cultures were incubated at 28 °C for 5 d in the dark. Then the mycelia of *A. flavus* were collected from YES cultures for the extraction of total RNA.

### Preparation of fungal total RNA, Illumina sequencing and bioinformatics analysis

The extraction of fungal total RNA, the preparation of cDNA libraries and RNA sequencing were conducted according to the methods described by Lv^35^. Total RNA was extracted with a Fungal RNA Kit (Omega, Norcross, GA, USA). The cDNA libraries were made using an Illumina^®^ TruSeq^TM^ RNA Sample Preparation Kit (Illumina Inc., San Diego, CA, USA) using an Illumina^®^ HiSeq 4000^TM^ system (Illumina Inc., San Diego, CA, USA). The clean reads were obtained by filtering the raw reads and used for subsequent analysis. Then they were mapped to the *A. flavus* genome, the EST sequencing and rRNA sequencing^33,35,76^, and assembled using programs TopHat 1.31, Bowtie and Cufflinks, respectively. The FPKM values were counted to calculate and normalize the transcription levels of genes in *A. flavus*^35,77^.

### Identification and analysis of differentially expressed genes

The difference in expression level between *A. flavus* genes treated with and without cinnamaldehyde was evaluated to be significant and a gene was identified as a differentially expressed gene when FDR value was ≤0.05^36^. For annotated genes, GO (gene ontology) functional analysis and KEGG (Kyoto Encyclopedia of Genes and Genome) pathway analysis were performed using FungiFun (https://sbi.hki-jena.de/FungiFun/FungiFun.cgi) and KAAS (KEGG Automatic Annotation Sever) annotation file, respectively^24,78–80^.

### RT-PCR and q-PCR analysis of aflatoxin biosynthesis genes

The isolation of RNA, synthesis of first-strand cDNA, RT-PCR and q-PCR were performed according to the methods described by Lv^35^. First-strand cDNA synthesis was carried out by RT-PCR using the Takara RNA Kit (AMV) ver. Q-3.0. (Takara Bio inc. Japan). All genes of aflatoxin cluster were analyzed. q-PCR was carried out using an ABI Prism 7500 Sequence Detection System (Applied Biosystems, Foster City, CA, USA).

### Availability of RNA-seq data

The raw RNA-Seq data of *A. flavus* discussed in this work have been deposited in the NCBI Sequence Read Archive with accession number of SRP132641.

## Supplementary information

Dataset 1
