# Supplementary material for: The anti-aflatoxigenic mechanism of cinnamaldehyde in Aspergillus flavus
Source: Sci Rep. 2019 Jul 19;9:10499. doi: 10.1038/s41598-019-47003-z (PMC6642104; doi:10.1038/s41598-019-47003-z)
Supplement: Supplementary file 1 — Dataset 1 [file 41598_2019_47003_MOESM1_ESM.docx]

Title:

The anti-aflatoxigenic mechanism of cinnamaldehyde in *Aspergillus flavus*

Author names and affiliations:

Ping Wang^1#^, Longxue Ma^1#^, Jing Jin^1^, Mumin Zheng^1^, Lin Pan^1^, Yueju Zhao^1^, Xiulan Sun^2^, Yang Liu^1^, Fuguo Xing^1^*

1. Institute of Food Science and Technology, Chinese Academy of Agricultural Sciences / Key Laboratory of Agro-products Quality and Safety Control in Storage and Transport Process, Ministry of Agriculture, Beijing 100193, P. R. China

2. State Key Laboratory of Food Science and Technology, School of Food Science, Synergetic Innovation Center of Food Safety and Nutrition, Jiangnan University, Wuxi, Jiangsu 214122, P. R. China

^#^ The authors contributed equally to this work.

*Corresponding Author

Institute of Food Science and Technology, Chinese Academy of Agricultural Sciences, 2 Yuanmingyuan West Road, Haidian District, Beijing 100193, P. R. China

Tel: +86-10-62811868 E-mail: [xingfuguo@caas.cn](mailto:xingfuguo@caas.cn)

Table S1 | The expression levels of genes in the biosynthesis of ergosterol

| gene ID (AFLA_x) | Untreated (FPKM) | R75 (FPKM) | LOG | annotated gene function |
| --- | --- | --- | --- | --- |
| 117780 | 34.58 | 72.15 | 1.06 | farnesyl-diphosphate farnesyltransferase, putative |
| 061500 | 215.97 | 199.72 | -0.11 | squalene monooxygenase Erg1 |
| 001030 | 0.16 | 1.76 | 0.16 | lanosterol synthase, putative |
| 006860 | 12.88 | 19.58 | 0.37 | oxidosqualene:lanosterol cyclase |
| 102160 | 8.15 | 14.00 | 0.78 | lanosterol synthase, putative |
| 036130 | 171.57 | 218.37 | 0.35 | 14-alpha sterol demethylase Cyp51A |
| 100590 | 18.36 | 18.72 | 0.03 | cytochrome P450, putative |
| 131060 | 68.63 | 105.48 | 0.62 | 14-alpha sterol demethylase Cyp51B |
| 051080 | 34.56 | 26.77 | -0.37 | c-14 sterol reductase |
| 111350 | 39.43 | 35.40 | -0.16 | C-14 sterol reductase, putative |
| 016870 | 41.14 | 46.19 | 0.17 | C-3 sterol dehydrogenase/C-4 decarboxylase |
| 088000 | 0.94 | 1.54 | 0.71 | NAD dependent epimerase/dehydratase, putative |
| 091760 | 9.40 | 6.47 | -0.54 | C-3 sterol dehydrogenase/C-4 decarboxylase family protein |
| 027490 | 7.19 | 16.38 | 1.19 | 3-ketosteroid reductase |
| 021770 | 7.19 | 16.38 | 2.08 | tocopherol O-methyltransferase, putative |
| 041230 | 4.14 | 17.47 | 0.57 | sterol 24-c-methyltransferase, putative |
| 110370 | 83.59 | 124.09 | -0.38 | S-adenosyl-methionine-sterol-C- methyltransferase |
| 029170 | 537.00 | 411.66 | 0.65 | sterol desaturase, putative |
| 018090 | 144.77 | 105.28 | -0.46 | sterol delta 5,6-desaturase ERG3 |
| 054820 | 112.96 | 143.14 | 0.34 | sterol delta 5,6-desaturase ERG3 |
| 028640 | 0.15 | 0.23 | 0.56 | cytochrome P450 sterol C-22 desaturase, putative |
| 035650 | 49.87 | 118.77 | 1.25 | cytochrome P450, putative |
| 022120 | 14.48 | 22.68 | 0.65 | c-24(28) sterol reductase |
| 078110 | 0.07 | 0.39 | 2.48 | C-14 sterol reductase, putative |
| 138060 | 2.02 | 2.68 | 0.41 | c-24(28) sterol reductase |

Table S2 | The expression levels of genes in the fatty acids *β*-oxidation /pentose phosphate pathway

| gene ID (AFLA_x) | Untreated (FPKM) | R75 (FPKM) | LOG | annotated gene function |
| --- | --- | --- | --- | --- |
| 019280 | 210.30 | 146.04 | -0.53 | peroxiredoxin, putative |
| 044460 | 1.07 | 1.19 | 0.15 | phenylacetyl-CoA ligase, putative |
| 086860 | 20.85 | 16.47 | -0.34 | NUDIX domain protein |
| 077250 | 6.43 | 6.74 | 0.07 | fatty-acyl coenzyme A oxidase (Pox1), putative |
| 052400 | 373.03 | 230.22 | -0.70 | isocitrate lyase AcuD |
| 033670 | 140.53 | 108.05 | -0.38 | lipid transfer protein, putative |
| 091060 | 1163.40 | 707.22 | -0.72 | allergen Asp F3 |
| 009410 | 1.16 | 0.53 | -1.15 | delta(3,5)-delta(2,4)-dienoyl-CoA isomerase, mitochondrial precursor, putative |
| 086800 | 116.77 | 103.81 | -0.17 | epoxide hydrolase, putative |
| 000640 | 2.31 | 2.20 | -0.07 | polyamine oxidase, putative |
| 134180 | 11.47 | 11.10 | -0.05 | peroxisomal 3-ketoacyl-coA thiolase (Kat1), putative |
| 070820 | 57.82 | 58.97 | 0.03 | 3-hydroxymethyl-3-methylglutaryl-Coenzyme A lyase, putative |
| 049390 | 146.01 | 126.51 | -0.21 | malate synthase AcuE |
| 134190 | 2.41 | 2.38 | -0.01 | bifunctional fatty acid transporter and acyl-CoA synthetase, putative |
| 018560 | 8.97 | 15.04 | 0.75 | ABC fatty acid transporter, putative |
| 073320 | 6.47 | 12.19 | 0.91 | 3-ketoacyl-CoA thiolase (POT1), putative |
| 079220 | 0.00 | 0.00 | / | glucose dehydrogenase, putative |
| 016570 | 2.48 | 4.54 | 0.87 | epoxide hydrolase, putative |
| 053060 | 130.76 | 123.92 | -0.08 | peroxiredoxin 5, prdx5, putative |
| 024060 | 2.38 | 1.73 | -0.46 | nonspecific lipid-transfer protein, putative |
| 135240 | 69.77 | 41.66 | -0.74 | carnitine acetyl transferase |
| 041590 | 40.07 | 47.55 | 0.25 | peroxisomal multifunctional beta-oxidation protein (MFP), putative |
| 090720 | 0.17 | 0.10 | -0.71 | male sterility domain containing protein, putative |
| 117410 | 0.06 | 0.00 | / | citrate synthase, putative |
| 026950 | 123.86 | 108.07 | -0.20 | 3-ketoacyl-coA thiolase peroxisomal A precursor |
| 041580 | 37.09 | 63.45 | 0.77 | estradiol 17 beta-dehydrogenase, putative |
| 115890 | 0.19 | 0.55 | 1.53 | acyl-CoA oxidase, putative |
| 099870 | 7.26 | 7.37 | 0.02 | alpha/beta hydrolase, putative |
| 100250 | 0.40 | 3.13 | 2.96 | catalase Cat |
| 018730 | 4.43 | 4.44 | 0.00 | epoxide hydrolase, putative |
| 015860 | 0.30 | 0.12 | -1.33 | aconitase, putative |
| 077250 | 6.43 | 3.79 | -0.76 | fatty-acyl coenzyme A oxidase (Pox1), putative |
| 077410 | 3.66 | 1.26 | -1.54 | acyl-CoA dehydrogenase, putative |
| 009410 | 1.16 | 0.76 | -0.62 | delta(3,5)-delta(2,4)-dienoyl-CoA isomerase, mitochondrial precursor, putative |
| 134180 | 11.47 | 9.40 | -0.29 | peroxisomal 3-ketoacyl-coA thiolase (Kat1), putative |
| 134190 | 2.41 | 2.23 | -0.11 | bifunctional fatty acid transporter and acyl-CoA synthetase, putative |
| 073320 | 6.47 | 8.86 | 0.45 | 3-ketoacyl-CoA thiolase (POT1), putative |
| 111190 | 24.06 | 18.83 | -0.35 | acyl-CoA dehydrogenase, putative |
| 041590 | 40.07 | 45.31 | 0.18 | peroxisomal multifunctional beta-oxidation protein (MFP), putative |
| 043970 | 3.61 | 2.62 | -0.46 | enoyl-CoA hydratase |
| 117410 | 0.06 | 0.08 | 0.57 | citrate synthase, putative |
| 026950 | 123.86 | 112.33 | -0.14 | 3-ketoacyl-coA thiolase peroxisomal A precursor |
| 041580 | 37.09 | 61.95 | 0.74 | estradiol 17 beta-dehydrogenase, putative |
| 115890 | 0.19 | 0.32 | 0.73 | acyl-CoA oxidase, putative |
| 080390 | 325.06 | 434.41 | 0.42 | *Sol*/6-phosphogluconolactonase, putative |
| 036840 | 529.55 | 705.44 | 0.41 | *Gnd1*/6-phosphogluconate dehydrogenase Gnd1, putative |
| 086620 | 515.87 | 656.01 | 0.35 | *Zwf1*/glucose-6-phosphate 1-dehydrogenase |

Table S3 | expression levels of Genes involved in development

| gene ID (AFLA_x) | Untreated(FPKM) | R75(FPKM) | LOG | annotated gene function |
| --- | --- | --- | --- | --- |
| 014260 | 2.51 | 17.01 | 2.76 | *RodB/HypB*conidial hydrophobin |
| 098380 | 12.35 | 89.35 | 2.85 | *RodA/RolA* conidial hydrophobin |
| 018340 | 46.12 | 82.85 | 0.84 | *GpaA/FadA* G-protein complex alpha subunit |
| 131330 | 46.54 | 53.47 | 0.20 | *NsdC* C2H2 zinc finger protein |
| 020210 | 86.15 | 144.00 | 0.74 | *NsdD* sexual development transcription factor |
| 026900 | 10.56 | 18.36 | 0.80 | *VosA* developmental regulator |
| 029620 | 1.02 | 3.18 | 1.64 | *AbaA* transcription factor |
| 033290 | 31.74 | 41.34 | 0.38 | *LaeA* regulator of secondary metabolism |
| 039530 | 9.85 | 3.98 | -1.31 | *FluG* family protein |
| 046990 | 148.93 | 289.53 | 0.96 | *StuA* APSES transcription factor |
| 052030 | 7.22 | 11.19 | 0.63 | *WetA*developmental regulatory protein |
| 066460 | 312.87 | 314.42 | 0.01 | *VeA* developmental regulator |
| 071090 | 893.92 | 588.41 | -0.60 | *EsdC* GTP-binding protein |
| 081490 | 36.78 | 42.74 | 0.22 | *VelB/Gda1*nucleoside diphosphatase |
| 074470 | 0 | 0 | / | *VelD/nuclear division Rtf1 protein, putative* |
| 082850 | 2.00 | 5.08 | 1.34 | *BrlA* C2H2 type conidiation transcription factor |
| 134030 | 10.32 | 18.82 | 0.87 | *FlbA* developmental regulator |
| 136410 | 89.17 | 140.06 | 0.65 | *Medusa* transcriptional regulator |

Table S4 | expression levels of Genes involved in MAPK pathway, oxylipins, and GPCRs

| gene IDAFLA_x | gene | Untreated(FPKM) | R75(FPKM) | LOG | annotated gene function | |  |  |
| --- | --- | --- | --- | --- | --- | --- | --- | --- |
| 062500 | *Maf1* | 66.38 | 69.76 | 0.07 | mitogen-activated protein kinase MAF1 | | | |
| 083380 | *Pbs2* | 35.91 | 47.70 | 0.41 | MAP kinase kinase (Pbs2), putative | | |  |
| 103480 | *Ste7* | 9.60 | 21.37 | 1.15 | MAP kinase kinase Ste7 | |  |  |
| 035530 | *Ste20* | 52.79 | 79.48 | 0.59 | serine/threonine kinase Ste20 | | |  |
| 048880 | *Ste11* | 13.04 | 22.29 | 0.77 | MAP kinase kinasekinase Ste11 | | |  |
| 021030 | */* | 25.45 | 29.71 | 0.22 | serine/threonine protein kinase, putative | | | |
| 052570 | *mpkA* | 44.75 | 71.08 | 0.67 | MAP kinase MpkA |  |  |  |
| 051240 | *Mkk2* | 100.19 | 127.98 | 0.35 | MAP kinase kinase (Mkk2), putative | | |  |
| 034170 | *Fus3* | 85.07 | 112.87 | 0.41 | MAP kinase FUS3/KSS1 | |  |  |
| 031560 | *bck1* | 12.93 | 21.96 | 0.76 | MAP kinase kinasekinase (Bck1), putative | | | |
| 100250 | *cat* | 0.40 | 3.13 | 2.96 | catalase Cat |  |  |  |
| 090690 | *Cat1* | 180.19 | 200.52 | 0.15 | mycelial catalase Cat1 | |  |  |
| 122110 | *cat2* | 20.23 | 20.19 | 0.00 | bifunctional catalase-peroxidase Cat2 | | | |
| 056170 | *catA* | 612.92 | 809.51 | 0.40 | spore-specific catalase CatA | | |  |
| 099000 | *sod1* | 98.10 | 124.30 | 0.34 | Cu,Zn superoxide dismutase SOD1 | | |  |
| 033420 | *mnSOD* | 1175.47 | 1318.19 | 0.17 | Mn superoxide dismutase MnSOD | | |  |
| 031340 | *atfA* | 227.96 | 223.53 | -0.03 | bZIP transcription factor (AtfA), putative | | | |
| 094010 | *atfB* | 212.65 | 279.64 | 0.40 | bZIP transcription factor (Atf21), putative | | | |
| 129340 | *ap-1* | 158.11 | 162.67 | 0.04 | conserved hypothetical protein | | |  |
| 110650 | *msnA* | 80.07 | 126.67 | 0.66 | C2H2 transcription factor (Seb1), putative | | | |
| 091490 | *mtfA* | 34.54 | 31.91 | -0.11 | C2H2 finger domain protein, putative | | | |
| 030580 | *pacC* | 129.52 | 151.82 | 0.23 | C2H2 transcription factor PacC, putative | | | |
| 034540 | *srrA* | 45.97 | 90.66 | 0.98 | stress response transcription factor SrrA/Skn7, putative | | | |
| 062210 | *sskA* | 22.66 | 37.22 | 0.72 | response regulator, putative | | |  |
| 068590 | *sskB* | 14.76 | 27.79 | 0.91 | MAP kinase kinasekinaseSskB, putative | | | |
| 061090 | *sakA* | 2.05 | 3.28 | 0.68 | MAP kinase SakA |  |  |  |
| 026790 | *ppoA* | 22.17 | 40.10 | 0.85 | fatty acid oxygenase PpoA, putative | | | |
| 120760 | *ppoB* | 0.49 | 1.42 | 1.54 | fatty acid oxygenase, putative | | |  |
| 030430 | *ppoC* | 1.04 | 5.45 | 2.39 | fatty acid oxygenase PpoC, putative | | | |
| 002850 | *AfPXG* | 395.51 | 334.87 | -0.24 | calcium binding protein Caleosin, putative | | | |
| 025100 | *gpdA* | 7357.45 | 4970.55 | -0.57 | glyceraldehyde 3-phosphate dehydrogenase GpdA | | | |
| 046760 | *gfdB* | 320.94 | 351.96 | 0.13 | glycerol 3-phosphate dehydrogenase (GfdB), putative | | | |
| 060740 | *gprA* | 11.82 | 8.59 | -0.46 | mating-type alpha-pheromone receptor PreB | | | |
| 061620 | *gprB* | 7.64 | 4.95 | -0.63 | a-pheromone receptor PreA | |  |  |
| 074150 | *gprC* | 2.56 | 7.19 | 1.49 | conserved hypothetical protein | | |  |
| 135680 | *gprD* | 10.67 | 5.78 | -0.88 | G protein-coupled receptor GprD | | |  |
| 006880 | *gprF* | 45.33 | 79.54 | 0.81 | PQ loop repeat protein | |  |  |
| 067770 | *gprG* | 40.30 | 28.63 | -0.49 | PQ loop repeat protein | |  |  |
| 006920 | *gprH* | 0.78 | 0.62 | -0.34 | cAMP receptor-like protein, putative | | | |
| 127870 | *gprJ* | 50.86 | 47.58 | -0.10 | vacuolar membrane PQ loop repeat protein | | | |
| 009790 | *gprK* | 0.25 | 0.72 | 1.50 | conserved hypothetical protein | | |  |
| 075000 | *gprM* | 3.33 | 15.84 | 2.25 | conserved hypothetical protein | | |  |
| 032130 | *gprO* | 40.06 | 39.76 | -0.01 | hemolysin-III channel protein Izh2, putative | | | |
| 088190 | *gprP* | 33.21 | 43.87 | 0.40 | IZH family channel protein (Izh3), putative | | | |
| 023070 | *gprR* | 32.65 | 38.94 | 0.25 | integral membrane protein | |  |  |
| 006320 | *gprS* | 12.95 | 18.69 | 0.53 | PQ loop repeat protein | |  |  |
| 117970 | *nopA* | 5388.07 | 5361.18 | -0.01 | opsin, putative |  |  |  |
